# Profiling the New Zealand Police Trainee Physical Competency Test

**DOI:** 10.3389/fpubh.2022.821451

**Published:** 2022-02-15

**Authors:** J. Jay Dawes, Jordan Scott, Elisa F. D. Canetti, Robert G. Lockie, Ben Schram, Robin M. Orr

**Affiliations:** ^1^School of Kinesiology, Applied Health, and Recreation, Oklahoma State University, Stillwater, OK, United States; ^2^Faculty of Health Sciences and Medicine, Bond Institute of Health and Sport, Bond University, Robina, QLD, Australia; ^3^Tactical Research Unit, Bond University, Robina, QLD, Australia; ^4^Department of Kinesiology, California State University, Fullerton, Fullerton, CA, United States

**Keywords:** law enforcement, PCT, occupational fitness, police academy, task performance

## Abstract

Police officers require a certain amount of occupational fitness to successfully perform physically demanding tasks. As such, trainees are required to undergo training to develop their ability to perform such tasks. The physical competency test (PCT) is a 400 m obstacle course consisting of key police occupational physical tasks used to evaluate a trainee's ability to complete tasks that a police officer is expected to perform whilst on duty. The purpose of this study was to profile the PCT in a police recruit population to provide an indication of the current level of occupational fitness within a policing population to inform conditioning requirements. Retrospective data for 813 male (age = 27.41 ± 5.92 years, body mass = 83.98 ± 14.03 kg, height = 179.23 ± 10.50 cm, BMI = 25.85 ± 3.92 kg/m2) and 372 female (mean age = 27.01 ± 6.45 years, mean weight = 67.14 ± 8.60 kg, mean height = 168.14 ± 6.46 cm and mean BMI = 23.61 ± 2.52 kg/m2) police trainees from the New Zealand Police Constabulary Recruitment database were provided for analysis. Anthropometric data, including height, body mass, and BMI were provided, in addition to trainee PCT time. Data were split by sex and age. Significant differences were observed between sexes for all anthropometric measures and PCT time (*p* < 0.001). Generally, in both the male and female groups, younger recruits tended to perform better than the older recruits with results between the “under 20” and the 20–24-year-old-age groups performing significantly better than the 35–39-year-old-age group in both sexes, and the 25–29-year-old-age group performing significantly better than the 35–39-year-old-age group in female officers. The data provided in this study provides a profile for performance of male and female recruits of various ages on the PCT in preparation for entry, or re-entry following injury, into the NZ Police. However, given that the PCT is considered a measure of occupational task performance, consideration should be given to the use of sex and age neutral requirements as the occupational tasks performed by police officers exhibit the same traits regardless of sex or age. Older trainees may therefore need conditioning to improve PCT times and subsequently occupational performance.

## Introduction

The primary role of police officers is to uphold the law and maintain peace in the community (1). Not only is policing a potentially dangerous occupation where officers may be required to respond to situations that threaten the health and well-being of themselves, colleagues, or other members of the community (2), it can also be physically and psychologically demanding (3). Despite components of an officer's job being sedentary (e.g., report writing, sitting in patrol cars or interviewing suspects or witnesses) (4, 5), there are intermittent periods which involve physically demanding tasks such as running, jumping, restraining perpetrators, self-defense, or manual handling tasks (5–7). These tasks must also be performed whilst carrying external loads including body armor and other vital equipment which can place additional stress on the body (8, 9).

Considering the nature of the aforementioned occupational tasks and the rapidly changing environment in which they are performed, police officers are required to have a high level of cardiovascular fitness, as well as muscular strength and endurance in order to be capable of performing physical tasks that arise (10, 11). Secondary to a demand for a high level of physical fitness, police trainees are typically required to undergo a series of physical tests prior to employment. Whilst these tests may be an indicator of future task performance (12), the primary aim is often to identify those at increased risk of injury during the training process (13, 14). Research has identified that police and military trainees who have lower levels of cardiovascular fitness are at increased risk of injury when compared to more physically fit members of their cohort (15–18). Similar findings have been reported in other studies, with reduced leg power, upper-body endurance, and grip strength being associated with an increased risk of injury (19, 20).

Fitness is thought to be associated with occupational performance. Orr et al. (20) found that increased grip strength was associated with greater performance in defensive tactics and marksmanship tasks among police trainees. Similarly, the findings of Bock et al. (21) indicated a possible association between decreased Functional Movement Screen scores and tactical operations task performance in police trainees. In specialist police, greater aerobic fitness has also been associated with improved load carriage performance (22). Likewise, fitness assessments such as push-ups and sit-ups have been commonly used to predict occupational performance (23). Lockie et al. (24) determined that the fitness characteristics that best related to performance in a Work Sample Battery Test (a job-specific fitness test including an obstacle course, body drag, fence climbs, and a 500-yard run) among law enforcement trainees were upper-body pulling strength, muscular endurance, anaerobic capacity and aerobic capacity. In contrast to these results, Carstairs et al. (25) discovered that push-ups and pull-ups were only correlated to one (a bridge building simulation) out of four army task simulations (pack lift and place, artillery loading, and M1 Tank loading). Due to the variability in predictive quality of the general fitness assessments listed above, attempts have been made to design occupation specific fitness assessments to increase the specificity of these assessments.

Previous research into the profiling of police officers has found that in general, both trainees and qualified officers tend to have greater levels of aerobic fitness than that of the average population (26). Similar studies have also found that younger trainees tend to outperform their older counterparts (5, 27). Despite numerous investigations exploring the relationships between fitness measures, injury and performance, minimal research has examined physically based work tasks. Therefore, the purpose of this study was to profile a Physical Competency Test (PCT) in a police trainee population.

## Methods

### Subjects

Retrospective data for 813 male (age = 27.41 ± 5.92 years, body mass = 83.98 ± 14.03 kg, height = 179.23 ± 10.50 cm, Body Mass Index (BMI) = 25.85 ± 3.92 kg.m2) and 372 female (age = 27.01 ± 6.45 years, body mass = 67.14 ± 8.60 kg, height = 168.14 ± 6.46 cm, BMI = 23.61 ± 2.52 kg.m2) trainee police officers were provided from the NZ Police Constabulary Recruitment database. This data included participant age, height, body mass, BMI and PCT score. All data were collected in a timeframe between 6 months and 8 weeks prior to the beginning of initial police training during 2016 to 2018 and was non-identifiable before analysis. Inclusion criteria for study subjects required participants be eligible to attend police college as well as attending police college after obtaining a passing grade on a Physical Appraisal Test (PAT). Prior to attempting the PCT, all trainees were required to complete a risk stratification questionnaire to identify individuals at risk of suffering from an adverse event during exercise. All trainees were also required to obtain medical clearance from a general practitioner prior to attempting the PCT. The Bond University Human Research Ethics Committee granted ethics approval for this study (BUHREC, Research Protocol BS02086). The typical participant consent process was waived as the data was retrospective and non-identifiable. Gatekeeper approval for use of the data was provided by the law enforcement agency from which this data was drawn.

### Physical Competency Test

The PCT is a 400 m obstacle course used to evaluate and assess a trainee's ability to perform occupation specific tasks which a police officer may have to perform whilst on duty (28). It has been shown to be a valid measure of the physical demands faced by frontline police officers (28). The course involves a series of 10 tasks performed in succession with the aim of finishing all tasks in the fastest time possible (29). Time is started at the commencement of the first task and stopped following the completion of all tasks when the trainee crosses the finish line. Trainees are given a pass-fail score based on the time for completion of the PCT, which is varied based on both age and sex. The tasks completed within the PCT are as follows:

Task 1: Trailer push: Trainees are required to push a 450 kg trailer 10 m as fast as possible. The trailer must be brought to a complete stop after 10 m prior to moving onto the next task.

Task 2: Wheel assembly carry: Trainees are required to remove a wheel from the trailer weighing ~22 kg, carry it 10 m and place the tire in a marked square.

Task 3: 200 m run: Trainees are then required to sprint 200 m to a balance beam.

Task 4: Balance beam: Trainees are required to negotiate a 5 m “L” shaped beam elevated 1 m above the ground and descend from the beam with a two-foot landing.

Task 5: 1.8 m long jump: With a running start, trainees were required to jump over a 1.8 m distance (marked on the ground) to mimic jumping over a ditch or obstacles during a foot pursuit.

Task 6: 1 m vault: This purpose of this task was to mimic the requirement of jumping over an obstacle during a foot pursuit. Trainees were required to run toward the 1 m high obstacle and vault over it with at least one foot planted on the hurdle during the task.

Task 7: 30 m agility run: Trainees were instructed to run around a series of six cones in a zig-zag fashion for 30 m. Following this, they were required to crawl under two small hurdles whilst getting up between each hurdle.

Task 8: Window climb and wall vault: Trainees had to run from the second hurdle to the window where they were instructed to climb through a window 1 m in height, after which they would run to a 1.8 m high wall and were instructed to vault over the wall.

Task 9: 75 kg body drag: This task was designed to imitate a victim drag during an emergency situation on duty. Trainees were required to lift and drag a 75 kg dummy whilst walking or running backwards for 7.5 m.

Task 10: Wire fence climb: The final task required trainees to climb a 2.2 m high wire mesh fence and completing the task with a two-foot landing. This was followed by a sprint to the finish line.

### Statistical Analysis

Data were presented digitally via a Microsoft Excel spreadsheet and prior to analysis was examined for accuracy and cleaned for any errors (e.g., missing data or improbable results [more than 2 standard deviations above the mean]). Data were then imported into SPSS (v23.0) for analysis, and presented as mean ± SD. A Shapiro Wilk test was performed to assess the normality and distribution of the data and a Mann-Whitney U test was performed to determine any differences between sexes with alpha levels set at *p* < 0.05. Additionally, a one-way analysis of variance (ANOVA) was performed to determine any differences of physical characteristics and PCT performance based on age with age in 5-year increments (under 20, 20–24, 25–30 years, etc.); an approach used in previous law enforcement research (12, 30). Alpha levels were set at 0.01 to account for data that were not normally distributed (31).

## Results

Results of the PCT times can be seen reported by sex and age group in Table 1. On average, males were significantly heavier (*p* < 0.001), taller (*p* < 0.001), and had a greater BMI (*p* < 0.001) than the females. Considering the pooled group of trainees, the males PCT time (1.54 ± 0.16 min) was significantly quicker (*p* = 0.001) than the females (2.21 ± 0.16 min). Males also had significantly faster PCT times than females in all age groups (*p* < 0.001).

**Table 1:** Profile of PCT results by sex in 5-year age increments.

**Age group (years)**	**PCT time (min: sec)**	
	**Females**	**Males**
Under 20 *n* = 29 (♀ = 11: ♂ = 18)	2.16 ± 0.03^†^	1.47 ± 0.16^*^^†^
20–24 *n* = 188 (♀ = 69: ♂ = 119)	2.17 ± 0.11^†^	1.50 ± 0.16^*^^†^^‡^
25–29 *n* = 159 (♀ = 40: ♂ = 119)	2.20 ± 0.10^†^	1.56 ± 0.16^*^
30–34 *n* = 78 (♀ = 19: ♂ = 59)	2.14 ± 0.16	1.55 ± 0.16^*^
35–39 *n* = 39 (♀ = 10: ♂=29)	2.41 ± 0.22	2.04 ± 0.09^*^
40–44 *n* = 25 (♀ = 10: ♂ = 15)	2.30 ± 0.22	2.04 ± 0.10^*^
45–49 *n* = 3 (♀ = 3: ♂ = 0)	2.28 ± 0.14	-

* *Significantly faster than the female PCT time of that age group (p < 0.001)*.

† *Significantly faster than the 35–39 Age Group of that sex (p < 0.01)*.

‡ *Significantly faster than the 40–44 age group of that sex (p < 0.001)*.

Upon comparison between female PCT performance and age, there were significant differences (*p* < 0.001) observed between the under 20 age and 20–24-year-old age groups and the 35–39-year-old age group with the younger officers outperforming the older. When comparing the 25–29-year-old age group, the only significant difference was seen with the 35–39-year-old age group (*p* < 0.001) which followed the same trend toward better performance in the younger officers. There was no significant difference seen between 30 and 34, 40 and 44, or 45 and 49-year-old age groups.

In the male population, the same trend was found with younger officers outperforming the older officers. Significant differences were seen between the under 20 age group and the 35–39-year-old age group (*p* = 0.008). In the 20–24-year-old age group, significant differences were seen between them and the 35–39-year-old age group (*p* < 0.001) and the 40–44-year-old age group (*p* = 0.009). Interestingly, no significant differences existed in the 25–29-year-old age group and whilst there were differences when comparing the 30–34-year-old age groups PCT performance with the other ages, these differences were not large enough to reach significance.

## Discussion

The purpose of this study was to profile the PCT in a police trainee population to provide an indication of the current level of occupational fitness within a policing population. On average, it was found that males tended to be taller, heavier, and have a greater BMI than female police trainees. Male trainees were also found to have significantly faster PCT times than female trainees across all ages. Through profiling these differences in PCT performance, an understanding of the differences in physical conditioning requirements by age and sex can be identified and catered for.

The results of this study support previous literature in which males were, in general, taller, and heavier than females (32). Similar findings have been reported in law enforcement research specifically (5, 33). Given the moderate (*r* = −0.515) to strong (*r* = 0.698) relationships between the PCT and the physical ability test (PAT: 2.4 km run, vertical jump, push-ups, and grips strength), it could be proposed that, in general, the males in this cohort were stronger, more powerful and had a high aerobic capacity than the females in this cohort (34, 35). One noted reason for this greater level of strength and power, both of which would be of importance in the tasks performed as part of the PCT (34), is that males typically possess greater lean body mass compared to females (23, 36, 37). The importance of lean body mass has been noted by Reilly et al. (37) who found lean body mass was significantly correlated with lifting and pulling tasks in a population of Canadian armed forces soldiers. Likewise, a study conducted by Dawes et al. (23) with 76 male police officers found that higher estimated lean body mass was significantly and positively correlated with push-ups, bench press and vertical jump performance. However, it should be noted that recent research by Kukic et al. (38) found that once BMI rose above 27.5 kg/m2 and Skeletal Muscle Mass Index above 14.10 kg/m2 (very muscular), performance on some tasks tended to be lower. As such, rather than focusing solely on the development of physical attributes (i.e., strength, power, aerobic fitness, etc.), consideration should be given to optimizing morphology (i.e., lean body mass).

Differences in lean body mass may also help explain the differences associated with aging. For example, Janssen et al. (36) reported a negative correlation between muscle mass with aging in both men and women. Similarly, in a population of 654 men and women aged 20–93 years, Lindle et al. (39) found that significant decreases in peak knee extensor torque existed with aging in males and females. These observed changes may be because of decreased growth hormone production (40, 41), androgens (40, 42) and insulin like growth factor (40, 42) as well as a reduction in muscle fiber cross sectional area (43) and body cell mass (44) with age. As such, it may not be surprising that when PCT performance was stratified by age, there would be significant differences between groups with a general progressive decline in completion times with age. These assumptions are also supported in the literature in a sample of state highway patrol officers (5) and military personnel (45). Thus, focusing on morphology in addition to physical attributes may be of benefit in the physical preparation of future police officers. Application of this concept would move beyond physical conditioning. For example, while strength training may improve both physical attributes (i.e., strength) and lean body mass (i.e., morphology), the inclusion of optimized diet may further contribute to morphological optimization.

However, this may not always be the case. For example, in a study of push-up performance by Dawes et al. (46) push-up performance was found not to deteriorate between age groups in a law enforcement population (20–59 year-old officers). While significant differences existed in this study between the 35–39-year-old age group and the four younger age groups in female trainees, no significant differences were seen between the 45–49-year-old age group or any of the younger age groups. These results were unexpected and may be attributed to a small sample size of three subjects in the 45–49-year-old age group, who may have been more physically fit in general. Similar results were seen in the male population. For instance, the under 20 years age group had significantly faster PCT times in comparison to 35–39 and 40–44-year-old age groups, however there were no significant differences observed between the 30–34-year-old age group and any of the younger or older age groups. As such, while there are general trends in physical performance associated with age (and sex), these are not all encompassing. As such, blanket approaches for groups (e.g., a group diet plan, or one-size-fits-all training program) may not be appropriate for large and diverse cohorts undergoing police recruit training.

Apart from morphological changes (i.e., decreased lean body mass), general declines in strength and power may also explain lower scores among older trainees, as these measures are important for optimal performance in the PCT and occupational tasks in policing in general (34). When investigating relationships between grip strength and PCT performance, Lockie et al. (34) found that left and right grips strength accounted for between 32% (right hand) and 45% (left hand) of the variance in PCT performance. This supposition between strength and power and physical occupational task assessments is further supported in wider literature. For example, where Izquierdo et al. (47) found that increasing age was associated with a significant decrease in squat jump, countermovement jump, and standing long jump performance, research by Dawes et al. (48) found that vertical jump height was correlated with sprinting performance in special weapons and tactics police officers. Similarly, Lockie et al. (24) found that greater lower limb strength was associated with improved performance in victim drag tasks. As such, age associated decrements in fitness could lead to decreases in police occupational task performance and result in slower PCT times. This surmise is supported by the findings of Perry et al. (49) who found that younger adults displayed greater knee and ankle isometric, concentric and eccentric strength, and power than older age groups. As sprinting and victim drag tasks are components of the PCT and given the aforementioned link between increased vertical jump height and sprint performance, lower limb strength with victim drag performance, the potential for decreases in PCT performance with aging is not unexpected.

Considering these sex and age-related differences, it is important to note that the occupational tasks required by police officers are sex and age neutral, being that a given task does not change with the sex or age of the officer. As such, typically, occupational assessments are based around the ability of a person to complete required occupational tasks regardless of sex or age (50). As an example, if the job requirement is to lift and carry a 13 kg artillery shell, the weight of the shell will not change with the lifter's sex or age. The importance of sex and age neutral assessments of task capability are typified by the Australian Army Physical Employments Standards—Army (PES-A) assessment (51) and the U.S. Army Occupational Physical Assessment Test (OPAT) (52). On this basis, while sex and age may influence task performance, if the task remains extant, then consideration should be given to the assessment criteria likewise remaining extant. For this to occur the validity of the PCT as a true measure of police task performance would need to be confirmed and a standard deemed to be suitable for the safe and effective completion of these tasks for all officers determined.

## Study Limitations

While this study provides greater insight into the occupational fitness levels of a police recruit population, it is not without limitations. Based on the archival nature of this data the investigators were limited to analysis of the variables provided by the agency. Future studies should investigate the relationships between general fitness tests and their relationships to the occupational tasks highlighted in this work. Having a better understanding of the underlying physical attributes that contribute to police task performance in these specific tasks could be used to develop individualized training strategies to maintain physical strengths, improve upon identified weaknesses, and inform return-to-work following injury reconditioning.

## Conclusion

The findings of this study suggest that, in general, male trainees completed the PCT faster than female trainees. Furthermore, younger police trainees across both sexes tended to perform better in the PCT in comparison to older trainees. The findings of this study highlight the need to consider sex and age-based attributes for the physical conditioning of trainees in addition to concepts beyond just physical training (like nutrition) to optimize performance. Older trainees may need further conditioning to improve PCT times to improve occupational performance and reduce injury risk. Additionally, the data provided in this study may be used to assist in further development of performance benchmarks for performance of male and female trainees of various ages on the PCT. However, consideration should be given to the development of sex and age neutral standards if the PCT is considered a measure of police task performance.

## Data Availability Statement

The datasets presented in this article are not readily available because the datasets generated in this study are not publicly available. They may be made available following approval by the law enforcement agency and by meeting ethics requirements. Please contact corresponding author should you require access to the data. Requests to access the datasets should be directed to rorr@bond.edu.au.

## Ethics Statement

The studies involving human participants were reviewed and approved by Bond University Human Research Ethics Committee (BS02086). Written informed consent for participation was not required for this study in accordance with the national legislation and the institutional requirements.

## Author Contributions

JD: methodology, data analysis, reviewing, editing, and finalizing manuscript. JS: investigation, initial data analysis, drafting, and project administration. RO, BS, and EC: conceptualisation, methodology, analysis, data curation, supervision, funding acquisition, and project administration. RL: data analysis, reviewing, editing, and finalizing manuscript. All authors contributed to the article and approved the submitted version.

## Funding

This work was funded by the New Zealand Police Force.

## Conflict of Interest

The authors declare that the research was conducted in the absence of any commercial or financial relationships that could be construed as a potential conflict of interest.

## Publisher's Note

All claims expressed in this article are solely those of the authors and do not necessarily represent those of their affiliated organizations, or those of the publisher, the editors and the reviewers. Any product that may be evaluated in this article, or claim that may be made by its manufacturer, is not guaranteed or endorsed by the publisher.

## References

[1] Meena JK Kumar R Meena GS. Protect the protector: morbidity and health behavior among police personnel in national capital region of India. Indian J Occupat Environ Med. (2018) 22:86. 10.4103/ijoem.IJOEM_28_18 30319229 PMC6176699

[2] Anderson GS Litzenberger R Plecas D. Physical evidence of police officer stress. Policing. (2002) 25:399–420. 10.1108/13639510210429437

[3] Shusko M Benedetti L Korre M Eshleman EJ Farioli A Christophi CA. Recruit fitness as a predictor of police academy graduation. Occupat Med. (2017) 67:555–61. 10.1093/occmed/kqx127 29016876 PMC6317156

[4] Violanti JM Ma CC Fekedulegn D Andrew ME Gu JK Hartley TA. Associations between body fat percentage and fitness among police officers: a statewide study. Safety Health Work. (2017) 8:36–41. 10.1016/j.shaw.2016.07.004 28344839 PMC5355530

[5] Dawes JJ Robin MO Flores RR Lockie RG Kornhauser C Holmes R. A physical fitness profile of state highway patrol officers by gender and age. Annals Occupat Environ Med. (2017) 29:1–11. 10.1186/s40557-017-0173-0 28588897 PMC5455072

[6] Lyons K Radburn C Orr R Pope R. A profile of injuries sustained by law enforcement officers: a critical review. Int J Environ Res Public Health. (2017) 14:142. 10.3390/ijerph14020142 28165373 PMC5334696

[7] Pryor RR Colburn D Crill MT Hostler DP Suyama J. Fitness characteristics of a suburban special weapons and tactics team. J Strength Conditioning Res. (2012) 26:752. 10.1519/JSC.0b013e318225f177 22289693

[8] Tomes C Orr R Pope R. The impact of body armor on physical performance of law enforcement personnel: a systematic review. Annals Occupat Environ Med. (2017) 29:1–15. 10.1186/s40557-017-0169-9 28515947 PMC5434519

[9] Dempsey PC Handcock PJ Rehrer NJ. Impact of police body armour and equipment on mobility. Appl Ergon. (2013) 44:957–61. 10.1016/j.apergo.2013.02.011 23668780

[10] Boyce RW Ciulla S Jones GR Boone EL Elliott SM Combs CS. Muscular strength and body composition comparison between the charlotte-mecklenburg fire and police departments. Int J Exerc Sci. (2008) 1:125–35.

[11] MacDonald D Pope R Orr R. Differences in physical characteristics and performance measures of part-time and full-time tactical personnel: a critical narrative review. J Military Veterans Health. (2016) 24:45.

[12] Lockie RG Dawes JJ Orr RM Stierli M Dulla JM Orjalo AJ. Analysis of the effects of sex and age on upper- and lower-body power for law enforcement agency recruits before academy training. J Strength Conditioning Res. (2018) 32:1968. 10.1519/JSC.0000000000002469 29401198

[13] Rappole C Grier T Anderson MK Hauschild V Jones BH. Associations of age, aerobic fitness, and body mass index with injury in an operational Army brigade. J Sci Med Sport. (2017) 20:S45–50. 10.1016/j.jsams.2017.08.003 28882434

[14] Reynolds K Cosio-Lima L Bovill M Tharion W Williams J Hodges T. A comparison of injuries, limited-duty days, and injury risk factors in infantry, artillery, construction engineers, and Special Forces soliders. Military Med. (2009) 174:702–8. 10.7205/MILMED-D-02-2008 19685841

[15] Pope RP Herbert R Kirwan JD Graham BJ. Predicting attrition in basic military training. Military Med. (1999) 164:710. 10.1093/milmed/164.10.710 10544625

[16] Jones BH Hauret KG Dye SK Hauschild VD Rossi SP Richardson MD. Impact of physical fitness and body composition on injury risk among active young adults: a study of Army trainees. J Sci Med Sport. (2017) 20:S17–22. 10.1016/j.jsams.2017.09.015 28993131

[17] Lockie RG Dawes JJ Orr RM Dulla JM. Physical fitness: Differences between initial hiring to academy in law enforcement recruits who graduate or separate from academy'. Work (2021) 68:1081–90. 10.3233/WOR-213438 33843714

[18] Hinton B Stierli M Orr RM. Physiological issues related to law enforcement personnel. In: Alvar B Sell K Deuster PA editors. NSCA's Essentials of Tactical Strength and Conditioning: Human Kinetics. Champaign, Ill (2017). p. 577–604.

[19] Orr R Pope R Peterson S Hinton B Stierli M. Leg power as an indicator of risk of injury or illness in police recruits. Int J Environ Res Public Health. (2016) 13:237. 10.3390/ijerph13020237 26907311 PMC4772257

[20] Orr R Pope R Stierli M Hinton B. Grip strength and its relationship to police recruit task performance and injury risk: a retrospective cohort study. Int J Environ Res Public Health. (2017) 14:941. 10.3390/ijerph14080941 28825688 PMC5580643

[21] Bock C Stierli M Hinton B Orr R. The functional movement screen as a predictor of police recruit occupational task performance. J Bodywork Movement Therapies. (2016) 20:310–5. 10.1016/j.jbmt.2015.11.006 27210848

[22] Robinson J Roberts A Irving S Orr R. Aerobic fitness is of greater importance than strength and power in the load carriage performance of specialist police. Int J Exerc Sci. (2018) 11:987–98. 30147827 10.70252/IWXE4027 PMC6102195

[23] Dawes JJ Orr RM Siekaniec CL Vanderwoude AA Pope R. Associations between anthropometric characteristics and physical performance in male law enforcement officers: a retrospective cohort study. Annals Occupat Environ Med. (2016) 28:26. 10.1186/s40557-016-0112-5 27293769 PMC4901472

[24] Lockie RG Dawes JJ Balfany K Gonzales CE Beitzel MM Dulla JM. Physical fitness characteristics that relate to work sample test battery performance in law enforcement recruits. Int J Environ Res Public Health. (2018) 15:2477. 10.3390/ijerph15112477 30404195 PMC6266286

[25] Carstairs GL Ham DJ Savage RJ Best SA Beck B Doyle TLA. A Box lift and place assessment is related to performance of several military manual handling tasks. Military Med. (2016) 181:258. 10.7205/MILMED-D-15-00070 26926751

[26] Maupin D Robinson J Wills T Irving S Schram B Orr R. Profiling the metabolic fitness of a special operations police unit. J Occupat Health. (2018) 60:356–60. 10.1539/joh.2018-0029-OA 29899197 PMC6176029

[27] Orr RM Pope R Stierli M Hinton B. A functional movement screen profile of an Australian state police force: a retrospective cohort study. BMC Musculoskeletal Disord. (2016) 17:296. 10.1186/s12891-016-1146-0 27431669 PMC4950801

[28] Handcock P Dempsey P. Fit to serve. a review of the New Zealand Police Physical Competency Test. J Sci Med Sport. (2011) 14:e56. 10.1016/j.jsams.2011.11.115

[29] New, Zealand Police. The PCT Test 2022. Available online at: https://www.newcops.govt.nz/file/pct-test.

[30] Bloodgood AM Dawes JJ Orr RM Stierli M Cesario KA Moreno MR. Effects of sex and age on physical testing performance for law enforcement agency candidates: implications for academy training. J Strength Conditioning Res. (2021) 35:2629–35. 10.1519/JSC.0000000000003207 31356509

[31] Lockie RG Dawes JJ Kornhauser CL Holmes R. Cross-sectional and retrospective cohort analysis of the effects of age on flexibility, strength endurance, lower-body power, and aerobic fitness in law enforcement officers. J Strength Condition Res. (2019) 29:2954–63. 10.1519/JSC.0000000000001937 28445229

[32] Haff GG Triplett NT. Essentials of Strength Training and Conditioning, 4th Edn. Human kinetics (2015).

[33] Orr R Dawes J Pope R Terry J. Assessing differences in anthropometric and fitness characteristics between police academy cadets and incumbent officers. J Strength Condition Res. (2018) 32:2632. 10.1519/JSC.0000000000002328 29120983

[34] Orr R Sakurai T Schram B Lockie R Dawes JJ. Relationships Between Physical Fitness Assessment Measures and a Workplace Task-Specific Physical Assessment: A Retrospective Cohort Study. Adelaide, SA: Australian Physiotherapy Association Conference (2019). 10.1519/JSC.0000000000004301 35836316

[35] Orr RM Canetti E Movshovich J Lockie R Dawes J Schram B. Profiling the New Zealand Police Physical Appraisal Test. (2021). 10.1108/IJES-06-2020-0032

[36] Janssen I Heymsfield SB Wang ZM Ross R. Skeletal muscle mass and distribution in 468 men and women aged 18–88 yr. J Appl Physiol. (2000) 89:81. 10.1152/jappl.2000.89.1.81 10904038

[37] Reilly T Spivock M Prayal-Brown A Stockbrugger B Blacklock R. The influence of anthropometrics on physical employment standard performance. Occupat Med. (2016) 66:576–9. 10.1093/occmed/kqw062 27261454

[38] Kukic F Cvorovic A Dawes J Orr RM Dopsaj M. Relations of body voluminosity and indicators of muscularity with physical performance of police employees: pilot study. Baltic J Sport Health Sci. (2018) 4:30–8. 10.33607/bjshs.v4i111.675

[39] Lindle RS Metter EJ Lynch NA Fleg JL Fozard JL Tobin J. Age and gender comparisons of muscle strength in 654 women and men aged 20–93 yr. J Appl Physiol. (1997) 83:1581. 10.1152/jappl.1997.83.5.1581 9375323

[40] Roubenoff R. Hormones, cytokines and body-composition—can lessons from illness be applied to aging. J Nutr. (1993) 123:469–73. 10.1093/jn/123.suppl_2.469 8429406

[41] Rudman D Kutner MH Rogers CM Lubin MF Fleming GA Bain RP. Impaired growth hormone secretion in the adult population: relation to age and adiposity. J Clin Invest. (1981) 67:1361. 10.1172/JCI110164 7194884 PMC370702

[42] Baumgartner RN Waters DL Gallagher D Morley JE Garry PJ. Predictors of skeletal muscle mass in elderly men and women. Mech Ageing Dev. (1999) 107:123–36. 10.1016/S0047-6374(98)00130-4 10220041

[43] Verdijk LB Snijders T Beelen M Savelberg HHCM Meijer K Kuipers H. Characteristics of muscle fiber type are predictive of skeletal muscle mass and strength in elderly men. J Am Geriatr Soc. (2010) 58:2069–75. 10.1111/j.1532-5415.2010.03150.x 21054286

[44] Kehayias JJ Fiatarone MA Zhuang H Roubenoff R. Total body potassium and body fat: relevance to aging. Am J Clin Nutr. (1997) 66:904. 10.1093/ajcn/66.4.904 9322566

[45] Dada EO Anderson MK Grier T Alemany JA Jones BH. Sex and age differences in physical performance: a comparison of Army basic training and operational populations. J Sci Med Sport. (2017) 20:S68–73. 10.1016/j.jsams.2017.10.002 29100826

[46] Dawes J Orr R Brandt B Conroy R Pope R. The effect of age on push- up performance amongst male law enforcement officers. Austral Strength Condition J. (2016) 24:23–27.

[47] Izquierdo M Aguado X Gonzalez R López JL Häkkinen K. Maximal and explosive force production capacity and balance performance in men of different ages. Eur J Appl Physiol Occupat Physiol. (1999) 79:260–7. 10.1007/s004210050504 10048631

[48] Dawes JJ Orr R Elder C Krall K Stierli M Schilling B. Relationship between selected measures of power and strength and linear running speed amongst special weapons and tactics police officers. J Austral Strength Condition (2015) 23:23–28.

[49] Perry M Carville S Smith I Rutherford O Newham D. Strength, power output and symmetry of leg muscles: effect of age and history of falling. Eur J Appl Physiol. (2007) 100:553–61. 10.1007/s00421-006-0247-0 16847676

[50] Orr RM Lockie R Milligan G Lim C Dawes J. Use of physical fitness assessments in tactical populations. Strength Condition J. (2021). 10.1519/SSC.0000000000000656

[51] Defence Science and Technology Organisation. Physical Standards for Military Service to Be Benchmarked. Media Release 043/2009 2009.

[52] Foulis SA Sharp MA Redmond JE Frykman PN Warr BJ Gebhardt DL. US army physical demands study: development of the occupational physical assessment test for combat arms soldiers. J Sci Med Sport. (2017) 20:S74–8. 10.1016/j.jsams.2017.07.018 28823473

